# Obesity and Cardiometabolic Risk Factors: From Childhood to Adulthood

**DOI:** 10.3390/nu13114176

**Published:** 2021-11-22

**Authors:** Dorota Drozdz, Julio Alvarez-Pitti, Małgorzata Wójcik, Claudio Borghi, Rosita Gabbianelli, Artur Mazur, Vesna Herceg-Čavrak, Beatriz Gonzalez Lopez-Valcarcel, Michał Brzeziński, Empar Lurbe, Elke Wühl

**Affiliations:** 1 Department of Pediatric Nephrology and Hypertension, Pediatric Institute, Jagiellonian University Medical College, 30-663 Cracow, Poland; 2 Pediatric Department, Consorcio Hospital General, University of Valencia, 46014 Valencia, Spain; alvarez_jul@gva.es (J.A.-P.); empar.lurbe@uv.es (E.L.); 3 CIBER Fisiopatología Obesidad y Nutrición (CIBEROBN), Instituto de Salud Carlos III, 28029 Madrid, Spain; 4 INCLIVA Biomedical Research Institute, Hospital Clínico, University of Valencia, 46010 Valencia, Spain; 5 Department of Pediatric and Adolescent Endocrinology, Pediatric Institute, Jagiellonian University Medical College, 30-663 Cracow, Poland; malgorzata.wojcik@uj.edu.pl; 6 Department of Medical and Surgical Sciences, University of Bologna-IRCCS S. Orsola, 40126 Bologna, Italy; claudio.borghi@unibo.it; 7 Unit of Molecular Biology and Nutrigenomics, School of Pharmacy, University of Camerino, 62032 Camerino, Italy; rosita.gabbianelli@unicam.it; 8 Department of Pediatrics, Pediatric Endocrinology and Diabetes, Medical Faculty, University of Rzeszów, 35-310 Rzeszów, Poland; drmazur@poczta.onet.pl; 9 Children’s Hospital Zagreb, Libertas International University, 10000 Zagreb, Croatia; vherceg@gmail.com; 10 Department of Quantitative Methods for Economics and Management, University of Las Palmas de Gran Canaria; 35017 Las Palmas, Spain; beatriz.lopezvalcarcel@ulpgc.es; 11 Department of Pediatrics, Gastroenterology, Allergology and Pediatric Nutrition, Medical University of Gdansk, 80-210 Gdansk, Poland; brzezinski@gumed.edu.pl; 12 Division of Pediatric Nephrology, Center for Pediatrics and Adolescent Medicine, Heidelberg University Hospital, 69120 Heidelberg, Germany

**Keywords:** obesity, cardiometabolic risk factors, hypertension, dyslipidemia, tracking phenomenon, nutrigenomics

## Abstract

Obesity has become a major epidemic in the 21st century. It increases the risk of dyslipidemia, hypertension, and type 2 diabetes, which are known cardiometabolic risk factors and components of the metabolic syndrome. Although overt cardiovascular (CV) diseases such as stroke or myocardial infarction are the domain of adulthood, it is evident that the CV continuum begins very early in life. Recognition of risk factors and early stages of CV damage, at a time when these processes are still reversible, and the development of prevention strategies are major pillars in reducing CV morbidity and mortality in the general population. In this review, we will discuss the role of well-known but also novel risk factors linking obesity and increased CV risk from prenatal age to adulthood, including the role of perinatal factors, diet, nutrigenomics, and nutri-epigenetics, hyperuricemia, dyslipidemia, hypertension, and cardiorespiratory fitness. The importance of ‘tracking’ of these risk factors on adult CV health is highlighted and the economic impact of childhood obesity as well as preventive strategies are discussed.

## 1. Introduction

The increasing prevalence of overweight and obesity, key components of the metabolic syndrome (MetS), in the pediatric and adult population poses a high risk of health complications and is associated with social and economic consequences.

According to the World Health Organization (WHO), the worldwide overall prevalence of obesity has nearly tripled since 1975. In 2016, 39% of adults aged 18 years and over were overweight and 13% were obese. However, among children and adolescents aged 5 to 19, the prevalence of overweight and obesity has risen even more dramatically: overweight increased from just 4% in 1975 to over 18% in 2016, while obesity increased from under 1% in 1975, to 6% in girls and 8% in boys. This is equivalent to more than 340 million overweight and 124 million obese children and adolescents worldwide in 2016 [1].

Obesity is associated with a high incidence of well-known cardiovascular (CV) risk factors such as dyslipidemia, hypertension (HTN) and diabetes. Numerous studies have shown the existence of a CV continuum, in which pathological processes begin as a result of various risk factors and lead to permanent changes and CV complications through endothelial damage, vascular and myocardial remodeling, and atherosclerotic processes. These changes may begin in early childhood and over time significantly increase the CV risk in young adults. This is of even greater concern in patients with already otherwise increased CV risk, e.g., in patients with chronic kidney disease (CKD), in whom the influence of classical and uremic risk factors is cumulative. The question arises, whether in the pediatric population early intervention in obesity will reduce the future CV risk in adulthood.

In this review we will discuss the importance of an early diagnosis and effective treatment of childhood obesity and the linked early and potentially reversible cardiovascular damage in children and adolescents to prevent CV complications, which remain the main cause of morbidity and mortality in the general population.

## 2. Cardiometabolic Risk Factors

### 2.1. Perinatal and Early Life Risk Factors

The concept that perinatal conditions, both intrauterine and early life increase the risk of many diseases later in life has gained traction over the last decades. The impact of early life on the development of obesity is a relevant issue due to its high prevalence and association with cardiometabolic risk factors. The greater propensity for obesity in later life seen in children heavier at birth, and an increase in central fat distribution in those with low birth weight (BW), suggest that fetal life is a critical window for programming later body adiposity. Scientific interest has grown regarding the associations of preconception, maternal and paternal health with childhood obesity. Furthermore, early child growth patterns have been emphasized as indicators of future child risks. The precise mechanisms of early programming of such disease states have only partially been elucidated; however, there is evidence to suggest that a window of opportunity may exist in the infant before and during pregnancy, and up to two years of age.

#### 2.1.1. Maternal Risk Factors

Epidemiologic and prospective cohort studies have identified maternal and gestational conditions that confer increased risk for subsequent cardiometabolic disorders [2].

Global rates of childhood obesity have increased dramatically. Evidence suggests that exposure in utero to maternal obesity or gestational diabetes mellitus (GDM) may contribute to these alarming trends [3,4]. Children born to mothers with GDM or obesity during pregnancy have an increased likelihood of developing obesity and metabolic disorders compared with unexposed children [3,4]. It is challenging to distinguish the effects of maternal obesity and diabetes in the preconception period compared to the gestational period, as these characteristics generally track over time. Effects and associated mechanisms could differ for preconception vs. gestational exposure, resulting in a complex interaction between the effects of both periods [5]. The epidemiological evidence supports the need for preconception and early-life interventions to reduce the obesity and diabetes burden in later life [6].

The potential role of the paternal metabolic contribution to a child’s later risk of disease has progressively gained more attention. Parental obesity is also a strong predictor of childhood obesity, and even more so when both parents are obese, this risk appears to be even greater [7]. Children of parents with obesity likely share not only genetic risks but also extra-uterine, environmental and lifestyle-related exposures that could explain some of the associations observed with parental preconception obesity and the offspring’s obesity risk.

There is some evidence that environmental exposures during pregnancy influence fetal growth and later risk of obesity on offspring. Maternal smoking during pregnancy is associated with restricted fetal growth [2]. Additionally, in later childhood, children of these mothers have a 1.5-fold greater risk for overweight and obesity as compared to those born to mothers who did not smoke [8]. Air pollution and exposure to synthetic chemicals occurring in utero and early childhood have been linked to effects on life-long risk of obesity and metabolic abnormalities [9].

The matter of artificial reproductive techniques (ART) and their impact on childhood obesity has received increased attention [10]. An increase in body fat in children born by “in vitro” fertilization was reported by Ceelen et al., as compared to the controls [11]. Although there is little data, the findings are sufficiently compelling to add ART to the list of prenatal risk factors related to long-term outcomes.

#### 2.1.2. Early Childhood Risk Factors

Birth weight (BW) is a sentinel marker of fetal health reflecting both, the intrauterine growth and the length of gestation. Not only does low BW merits consideration but also high BW which is a consequence of intrauterine overnutrition. Based on recent findings, the associations of abnormal fetal growth with heightened risk for cardiovascular and metabolic disease extend across a range of birth weights and postnatal growth patterns [9].

Early childhood growth trajectories and rapid catch-up growth have been shown to influence the development of risk factors. The impact of BW and postnatal growth on the presence of overweight or obesity, blood pressure (BP) values, metabolic parameters, HTN and type 2 diabetes (T2D), was assessed in a systematic review [12]. Some studies had a significant association with BW and/or postnatal growth while others did not. The most frequent association was seen with BP values and fasting insulin, with the greatest adverse levels present in those who were born with low BW but then became relatively heavy [12]. In one meta-analysis, the odds ratio for overweight and obesity was 3.66 [95% CI 2.59–5.17] in the presence of rapid weight gain before 2 years of age, even higher odds were observed when rapid weight gain occurred before 1 year of age [13].

The above findings underscore the importance of regular growth monitoring. The use of obesity criteria to identify children at risk will miss many at-risk persons. An upward crossing of BMI percentiles during childhood, that does not necessarily connote childhood obesity, also increases the risk.

Early-life feeding practices such as being breastfeed or not, timing of introduction and type of complementary feeding along with the association with obesity has been analyzed in observational studies. Some suggest a lower prevalence of overweight and obesity in children who were fed breast milk compared with infant formula, however, optimal duration of breastfeeding to provide substantial benefits remains unknown [14].

#### 2.1.3. Molecular Techniques and Their Contribution to Understanding Programming

Even when the precise mechanisms of early programming of such diseases later in life has not yet been fully understood, molecular studies can offer the opportunity to dive in depth. The molecular era allowed for the capacity to perform rapid nucleic acid sequencing and microarray studies, as well as other molecular techniques and has provided the potential for far more understanding of developmental origins of health and disease (DOHaD) [9].

There is now a growing literature concerning the role of epigenetics in DOHaD, the majority of which has been carried out in experimental models, particularly in rodents. The degree of adiposity in adult life has been correlated with changes in methylation of DNA at birth or early in life, suggesting that epigenetic markers might be sought as a predictor [15].

The microbiome and its contribution to health and disease have recently been of interest to the scientific community. It has been suggested that the microbiome influences postnatal programming and obesity. Increasingly, the concept that it may play a role in DOHaD seems appealing [16].

### 2.2. Diet as a Risk Factor for Obesity and Cardiovascular Complications

There is no doubt that a high intake of *sugar* causes weight gain, and therefore is associated with the development of obesity, insulin resistance, and dyslipidemia. All of them are the most important risk factors for the development of HTN and CV disorders. The analysis of the data from the National Health and Nutrition Examination Surveys (NHANES) 1988–1994, 1999–2004, and 2005–2010 (*n* = 31,147) revealed a significant direct relationship between added sugar consumption and increased risk for CV morbidity and mortality in adults [17]. Most of the added sugar are glucose, fructose, and sucrose (in the intestine sucrose is broken down into glucose and fructose, which are absorbed as such). Glucose excess is directly associated with hyperinsulinemia, while fructose excess increases uric acid levels and very-low-density lipoprotein (VLDL) levels leading to hyperuricemia and liver steatosis [18]. Moreover, it has been shown, that simultaneous consumption of sugar and salt by obese patients is a greater risk factor for developing high blood pressure than consuming each of them separately [19].

‘Salt’, properly sodium chloride (NaCl) is the main form of sodium intake. It is added to most of the processed food. Additionally, the source of sodium may be different food additives, such as sodium bicarbonate or sodium nitrate added to bread and meat products respectively [20]. The relationship between salt consumption and arterial HTN has been known since the beginning of the 20th century [21]. According to the traditional model, the relationship between sodium excess and the development of HTN in obese individuals is based on the increase of extracellular fluid volume and higher blood flow in the kidney [22]. High glomerular filtration rate and renal blood flow increase renal sodium reabsorption. Glomerular hyperfiltration and neurohumoral activation, within activation of the sympathetic nervous system, lead to the development of severe HTN, subsequent glomerular injury, and impaired renal sodium excretion capacity, resulting in the gradual loss of nephron function [22]. According to that classical theory of ‘salt sensitive’ arterial HTN development, massive salt restriction should be of universal benefit. On this basis, recommendations were made for general salt reduction [23]. More recent studies, however, show that dietary salt restriction only has a beneficial effect in certain groups, including patients with obesity and/or with HTN [24,25]. Moreover, not only sodium dose, but its ratio to potassium intake might be important. High Na^+^/K^+^ ratio intake is considered to be a stronger risk factor of HTN and cardiovascular disease than each of these nutrients alone [26]. Contrary, the Na^+^/K^+^ ratio positively affects the physiological rise of BP in childhood, resulting in smaller BP slopes [26]. Potassium increases urinary sodium excretion which diminishes total sodium content. In addition, potassium is thought to induce vascular smooth muscle relaxation and thus decrease peripheral resistance [27]. Therefore, the so-called DASH (Dietary Approaches to Stop Hypertension) diet, rich in fruits and vegetables, low-fat dairy products, and low saturated and total fat, is an effective tool in the treatment of HTN. It helps not only to reduce sodium load, but also to increase potassium intake.

Uric acid is the final product of purine metabolism in humans, and its production is largely dependent on the activity of xanthine-oxidoreductase (XOR) that is responsible for the final steps of the conversion of xantine to uric acid [28]. The serum levels of uric acid are also influenced by the renal excretion rate of urate that is controlled by renal tubular transport systems (mainly URAT-1 and GLUT-9) that are more directly involved in the global handling of uric acid metabolism [29]. The activation of XOR is leading to an increase in the production of pro-oxidative compounds that have been reported to be involved in the development of HTN, insulin resistance, MetS, diabetes as well as CV and renal disease [30,31]. In children, the increase in serum urate levels has been proven to be responsible for a significant increase in body weight and BP values that can contribute to cardiometabolic abnormalities early in life [32]. In particular, a close correlation has been reported between the fructose intake (a well-known precursor of uric acid) and the increase in body mass index (BMI) in US children and adolescents with a negative impact on the natural history of HTN and MetS [33]. According to the evidence provided by the Bogalusa Heart study [34] the increase in uric acid can antedate and predict the increase in body weight and BP values in childhood suggesting a primary pathogenetic role for elevated uric acid and/or for the mechanisms involved in its production (e.g., XOR activity). This has been recently confirmed by some interesting studies based on the analysis of temporal trajectories of serum urate and showing and increase in cardiac and metabolic disorders in those subjects bearing serum urate levels persistently elevated or progressively increase while the opposite was observed in subjects with serum urate levels persistently normal or undergoing a progressive reduction [35]. Thus, an increase in serum urate levels in children and adolescents may contribute to promote overweight and CV risk factors with a negative prognostic implication on the risk of cardiometabolic disease later in life.

### 2.3. Nutrigenomics and Nutri-Epigenetics

The nutrigenomics-impact of dietary components starts early in life and can influence health and disease across life. Dietary components are used to produce metabolites that can impact gene expression directly or through epigenetic mechanisms. Functional groups (i.e., methyl-, acetyl-, phosphate-group, etc.) able to modulate gene expression without any changes in DNA sequence, can be synthesized by dietary components [36]. Folate, vitamins (B12, B2, B6) betaine and choline are necessary to synthesize S-adenosine methionine (SAM), the universal methyl group donor, by one carbon cycle. The availability of methyl groups depends on a folate-rich diet (i.e., green leaves, peas, beans, lentils, liver, etc.) and on folic acid supplementation during pregnancy. B12 is present only in animal food (i.e., eggs, meat, fish), so vegans need oral sublingual treatment (to avoid its hydrolysis by the liver). Reduced level of substrates required for one carbon cycle during pregnancy, has been associated to impaired DNA methylation at promoter level of genes that regulate growth and metabolic diseases (i.e., IL10, LEP, ABCA1, GNASAS and MEG3) in siblings [37]. Differences in DNA promoter methylation (i.e., INSIGF, LEP and GNASAS) were measured also according to sibling gender and gestational timing of the malnutrition, and were associated with increased risk in developing obesity in men and glucose intolerance in women later in life. DNA methylation has a key role in the cell differentiation and the methylation of carbon 5 at the cytosine of CpG island of promoter region is associated to gene silencing [38]. Furthermore, an epigenetic memory of multiple environmental conditions (i.e., diet, air pollution, noise pollution, life style, work, family, education, physical activities, etc.) acquired during early life, remains longer and it can influence adult phenotype as well as be epigenetically inherited from generation to generation [39].

High fat diet, low or high protein intake can address epigenetic responses associated to metabolic diseases (i.e., cardiovascular diseases (CVD), T2DM, obesity, HTN, etc.) in adulthood [40]. The nutrigenomic impact of dietary lipids has been extensively studied [41]. Longitudinal analysis shows that chronic uses of high dietary saturated fatty acids (SFAs) or/and sugars increase the risk to develop CVD [42]. Exposure to palmitic acid, a SFA contained in several types of food (i.e., meat, eggs, butter, palm and coconut oils etc.,) is able to activate inflammatory responses by inflammasome activation (i.e., NLRP3, IL-1β, IL-18), while this effect was not observed after monounsaturated fatty acid exposure with oleic acid [43]. Overall, the consumption of ultra-processed food, rich in fats and sugars, low in fiber and antioxidant/anti-inflammatory bioactive compounds, was associated with a rise of caloric intake (about 500 Kcal/day) [44] and with an increase of risk of CVD and all-causes mortality [45]. These findings should serve as an incentive for limiting consumption of ultra-processed food, and encouraging natural or minimally processed foods, as several national policies recommend.

Furthermore, nutrigenomic impact of high protein intake has been investigated; high protein intake (more than the recommended daily intake (RDI) of 0.8 g/Kg per day) increases the risk of prediabetes and T2DM [46]. In addition, the source of proteins is important; Mediterranean, vegan or whole-food plant-based diets significantly increase high-density lipoprotein cholesterol (HDL-C) and may reduce incidence and mortality of CVD. Adherence to the Mediterranean diet has been associated with a metabolic signature useful to predict CVD risk [47], and new epigenetic biomarkers have been identified to predict the risk and the severity of CVD [48].

Additionally, not only strict vegetarian diets, but also a less strict plant-based diets with limited animal products have been associated with low systolic and diastolic BP [49]. A large prospective cohort study has demonstrated that the replacement of animal protein with plant protein (3% of the energy) decreases the CVD mortality (risk reduction 11% in men and 12% in women) [50]. The positive effect of plant-based food is associated to the nutrigenomic activity (i.e., antioxidant, anti-inflammatory, etc.) of polyphenols contained in plants, the positive impact of fiber on gut microbiota richness and diversity, the folic acid and the essential fatty acid content [51].

In summary, nutrigenomics and nutri-epigenetics outcomes have demonstrated how and when animal and plant-based food contribute to influence molecular responses (with beneficial or detrimental effects), supporting the positive role of high adherence to the Mediterranean and/or plant-based diet to reduce the risk to develop CVD. Strategies for prevention should take into account also the inheritance of epigenetic biomarkers across generations.

### 2.4. Dyslipidemia, Insulin Resistance, Hypertension and Cluster of CV Risk Factors

Dyslipidemias are disorders of lipoprotein metabolism that can result in the following abnormalities: high total cholesterol (TC), high low-density lipoprotein cholesterol (LDL-C), high non-high-density lipoprotein cholesterol (non-HDL-C), high triglycerides (TGs), and low HDL-C [52]. Lipid levels vary by age and sex and reference lipid and lipoprotein values have been derived from the population-based Lipid Research Clinical Prevalence Study, which obtained between 1972 and 1976 fasting lipoprotein profiles in more than 15,000 children and adolescents (age range 0 to 19 years), and from the United States National Health and Nutrition Examination Surveys (NHANES), which analyzed lipid levels in 7000 children between 1988 and 1994 [53,54,55,56]. In most patients with hyperlipidemia this condition is caused by some underlying “non-lipid” etiology rather than by a primary disorder of lipoprotein metabolism. Among the CV risk factors, lipids and lipoproteins are of special importance and in many studies, childhood obesity has been shown to be associated which increased levels of TC, LDL-C and TGs and decreased level of HDL-C [57,58]. CVD is the number one cause of death in the United States and 38% of adults affected by CVD have risk factors such as elevated serum lipid levels, diabetes, and high blood pressure. Many studies have confirmed an additional role of body fat distribution and in particular of excess visceral fat even in the adolescent age group [59,60].

Atherosclerosis can start at young age, and the number of young individuals developing atherosclerosis is on the rise, especially in children with risk factors such as familial hypercholesterolemia (FH), type 1 diabetes mellitus, chronic kidney disease and HTN. Furthermore, many studies have identified dyslipidemia as a risk for premature atherosclerosis, even in children and adolescents. In the Bogalusa Heart Study, autopsy studies performed in 204 young subjects demonstrated fatty streaks in 50 percent of cases between 2 and 15 years of age and in 85 percent of older subjects between 21 and 39 years of age [61]. The prevalence of raised fibrous plaques in the aorta and coronary arteries also increased with age from approximately 20 percent in subjects between 2 and 15 years of age to 70 percent in those between 26 and 39 years of age. The prevalence and the extent of atherosclerosis found in the aorta and coronary arteries were greater with increasing BMI, BP, and levels of serum TC and LDL-C. The degree of atherosclerotic changes increased with worsening severity and greater numbers of risk factors [62].

Similarly, the Pathobiological Determinants of Atherosclerosis in Youth (PDAY) study [63] reported raised fatty streaks in 10 percent of coronary arteries and 30 percent of aortas in subjects aged 15 to 19. The extent of fatty streaks increased with increasing age, elevated BP, higher serum LDL-C, and lower serum HDL-C. Female patients lagged by five years behind male patients in the progression of the extent of raised lesions in the right coronary arteries. In a subsequent report, individuals with early and more severe atherosclerotic changes were more likely to have had one or more CVD risk factor (including dyslipidemia, obesity, hyperglycemia, HTN, or smoking) [64].

Several longitudinal studies reported tracking of adverse lipid levels from childhood to adulthood. In a cohort of 725 adults (age range 33 to 42 years) in the Muscatine Study, childhood TC levels positively predicted adult carotid intima-media thickness (cIMT). In women, childhood BMI was also a significant predictor of cIMT [65,66]. In the Cardiovascular Risk in Young Finns study, elevated childhood levels of LDL-C and insulin, as well as obesity, were predictive of increased cIMT (observation period 27 years). In subsequent studies, carotid artery elasticity decreased as the number of childhood CVD risk factors increased and flow-mediated dilation was lower in male patients who had elevated BP during adolescence. In this cohort, exposure to CVD risk factors over time correlated with the extent of coronary artery calcification by computed tomography [67]. Similarly, in a cohort of patients involved in the CARDIA study, initially recruited at age 18 to 30 years and followed for 15 years, baseline CVD risk factors (smoking and higher LDL-C, glucose, and systolic blood pressure levels) were associated with increased risk of coronary artery calcium later in life [68]. The International Childhood Cardiovascular Cohort Consortium has performed a meta-analysis that combined data from the four prospective studies mentioned above. In this analysis, the number of childhood CVD risk factors (e.g., increased cholesterol, TGs, BP, and BMI), even in children as young as nine years of age, was predictive of elevated adult cIMT with progressive strengthening of the association through adolescence [69]. Further studies have shown that dyslipidemia in adolescence predicts increased adult cIMT, even after accounting for sex, obesity, and HTN. Screening of lipid levels in children may reveal both genetic lipid abnormalities (e.g., including familial hypercholesterolemia, which affects 1 in 250 people), and dyslipidemia, which responds favorably to lifestyle changes [70]. Even a small weight loss is associated with a significant decrease in the concentration of TG and an increase in the concentration of HDL-C. In addition to the recommended diet, physical activity (PA) is strongly recommended. According to the National Heart, Lung and Blood Institute (NHLBI) recommendations, in patients in whom non-pharmacological management has no effect, the use of lipid-lowering drugs should be considered [71].

The relationship between obesity and its complications, in particular insulin resistance, and arterial hypertension was first noticed in the 1950s [72]. Many studies over the years have confirmed that excess of fat tissue regardless of age and gender is associated with an increase of blood pressure [73,74,75]. Among children with obesity, the prevalence of arterial HTN is up to 30%, in contrast to less than 3 (5) % in the normal weight pediatric population [76,77,78,79] and weight gain is accounted for up to 75% of the risk for primary HTN [80].

The pathogenesis of HTN in obese individuals is complex and still not fully understood. Currently, it is believed that the abnormally increased activity of adipose tissue in the production of hormones and adipokines is of key importance. Pro-inflammatory substances as tumor necrosis factor-α, interleukin-6, C-reactive protein may lead to macrophage recruitment, which could increase pathological lipolysis. Subsequently excess lipid delivery could promote ectopic lipid accumulation leading to the associated impairments in insulin signaling, mainly in the liver and skeletal muscles, that in turn may also contribute to development of insulin resistance [81]. Insulin resistance and hyperinsulinemia are independent activators of the sympathetic nervous system. Other factors with a documented independent role in activating sympathetic nervous system in obese individuals are: leptin excess and intermittent hypoxia caused by sleep-disordered breathing [82,83,84]. The enhanced activity of sympathetic nervous system causes vasoconstriction and reduced renal blood flow, which is a trigger for renin release, and subsequent activation of the renin–angiotensin–aldosterone system (RAAS) results in sodium and water retention [82]. Additionally, it causes β2-adrenergic receptors dependent activation of the NaCl-cotransporter in the distal tubule, that is considered to be one of the most important mechanisms for the development of salt-sensitive HTN [85]. Although the activation of the RAAS has been well documented in adults and in experimental models, data regarding the role of this mechanism in the development of HTN are contradictory [86]. Interestingly, not only does the classic way of activating the RAAS play an important role in the development of obesity-related arterial HTN, but also plasma aldosterone concentration seems to be positively correlated with the amount of visceral adipose tissue, independent of plasma renin activity [87,88]. Contrary to lean hypertensive subjects, patients with obesity show a positive paradoxical correlation between sodium intake and aldosterone levels. It has been suggested, that some adipokines, as yet unidentified, may directly stimulate aldosterone release from adrenals in angiotensin II-independent manner [87,88]. It has also been proven that adipose tissue can produce angiotensinogen, angiotensin, and angiotensin II itself, stimulating aldosterone secretion by adipocytes in a paracrine/autocrine way independently from the inhibitory effect of high salt consumption [88,89]. Moreover, the results of genetic studies of humans suggest the association of obesity-related HTN with the variants of several genes involved in aldosterone secretion and metabolism, such as glucocorticoid receptor, aldosterone synthase (CYP11B2), and serum and glucocorticoid-regulated kinase 1 [90,91,92]. Variants of the latter are described to be associated with predisposition to HTN, hyperinsulinism and high salt intake [92]. An additional element may be the excessive stimulation of renin production in vitamin D deficiency, a condition often found in obese people [93,94]. Finally, cortisol production by adipose tissue may stimulate renin production exerting aldosterone-like effects through its mineralocorticoid activity and, moreover, may increase insulin resistance [95]. Undoubtedly, aldosterone is not only a hormone that regulates electrolytes and fluid volume, but can be an important mediator of obesity development independently of calorie intake and target-organ damage. Excess of aldosterone contributes to insulin resistance, and leptin resistance [96]. In the kidney, aldosterone causes podocyte injury, which leads to proteinuria and glomerulosclerosis, and proinflammatory responses, mediating perivascular and interstitial fibrosis [97,98].

Hyperinsulinemia has a similar effect leading to direct kidney damage by impairment in insulin metabolic signaling resulting in reduced NO production, associated impairment of tubuloglomerular feedback, and subsequently hyperfiltration and sodium retention [99]. Hyperinsulinemia is also directly related to the reduction in uric acid excretion. In a number of clinical trials, such as NHANES I, the Framingham Study and the Bogalusa Heart Study (including the pediatric population), it was shown that serum uric acid concentration is an independent prognostic factor for the development of arterial HTN [34,80,100]. Hyperuricemia causes renal vasculitis by the stimulation of nuclear transcription factors, release of pro-inflammatory cytokines, and pre-glomerular arteriolopathy due to e.g., increasing the proliferation of vascular smooth cells and causing inflammation and tubulointerstitial fibrosis. These changes further activate the RAAS and may additionally favor the adverse effect of urates on the glomerular vessels [82]. Additionally, direct compression of the renal parenchyma by perinephric fat may reduce intrarenal blood flow and increase sodium reabsorption, leading to volume expansion, increase of cardiac output and decrease in blood flow reserve [82]. This phenomenon occurs even in the absence of signs of glomerular sclerosis or chronic kidney disease [82]. While in the early stage of obesity induced HTN, the increased glomerular filtration rate and renal blood flow induce an increase in renal sodium absorption, with prolonged HTN, renal vasodilation, glomerular hyperfiltration, and neurohumoral activation led to further increase of BP, glomerular injury, and an impaired renal capacity for sodium excretion, resulting in the gradual loss of nephron and kidney function. In this way, obese subjects require a higher BP than lean subjects to maintain the sodium balance, indicating impaired renal-pressure natriuresis (‘salt-sensitive’ HTN) [86]. Also, pro-inflammatory substances formed in adipose tissue play a direct role in endothelial damage and increased vascular stiffness. Since HTN itself is also a factor leading to endothelial pathology a “vicious circle” phenomenon should be taken into account. With respect to the above-mentioned mechanisms of the development of HTN in obese children, it seems obvious that the basis of treatment and prevention of complications should be effective reduction of adipose tissue [101,102]. Limiting salt intake is a crucial element in the treatment of obesity-associated HTN. If pharmacotherapy has to be introduced, first-line drugs are antagonists of the RAAS (angiotensin-converting enzyme inhibitors, angiotensin receptor blockers [101,102], but also mineralocorticoid receptor antagonists may be considered in selected cases [103,104]).

### 2.5. Obesity or Cardiorespiratory Fitness—What Does Really Matter?

Cardiorespiratory fitness (CRF), also known as cardiorespiratory endurance, cardiovascular fitness, aerobic capacity, or aerobic fitness, refers to the “capacity of the circulatory and respiratory systems to supply oxygen to skeletal muscle mitochondria for energy production during PA” [105,106]. This is only 1 of 4 distinct health-related fitness components (CRF, muscular fitness, flexibility and body composition). Although often confused, PA and CRF are related but distinct concepts. “PA is voluntary movement produced by skeletal muscles that results in energy expenditure” [106], while “Exercise refers to a subset of PA in which the goal is to improve performance, health, or both” [106]. While CRF is having the capacity to perform or not perform a certain type of PA, PA is an action or behavior.

Over the last few years, CRF has acquired special scientific interest in the evaluation of youth´s health because it has been shown that it is a predictor of various indicators such as cardiometabolic health [107,108], premature CVD [109], academic achievement [110], and mental wellness [111]. In a cohort of overweight, obese, and control participants, Redon et al. concluded that CRF was inversely related with fasting insulin and the HOMA index, which is considered as a fingerprint for future metabolic disease [112]. In a systematic review and meta-analysis, low CRF in children and adolescents was notably associated with the development of metabolic syndrome [113].

CRF is able to be measured objectively and it can be tracked over time and compared over different populations [114]. Even though the cardiopulmonary exercise test (CPETs) is considered the gold standard method to evaluate CRF, it is not easily implemented [105]. There are also questionnaires designed for examining CRF in youth, but they are only recommended for epidemiological studies and not for estimating CRF in individuals [115]. As a result, outdoor or field procedures have been conveniently developed, among them, the 20m shuttle run test and the Cooper test [116,117] which are the most common. Additionally, there are some suitable tests for use in office settings such as the 6-Minute Walk Test and the Step Test. The Step Test could be an alternative to CPETs in order to estimate office-CRF, because it is easy to administer in limited indoor spaces and requires minimal equipment and training to be implemented [118].

CRF in youth is affected by non-modifiable factors as genetics, age, sex, race/ethnicity and prematurity and by modifiable factors as habitual PA and training, sedentary time, diet, social-economic-environmental factors and obesity. Many obese children and adolescents meet these modifiable factors, and it is shown that youth with obesity have lower CRF than their normal-weight peers [119]. Nevertheless, there is evidence that improvement in CRF in obese children and adolescents increases CV health, even among those who do not improve their body composition [120]. Moreover, overweight children and adolescents with a high fitness level (fat-but-fit subjects) have a healthier CV profile than their overweight, low fit peers and a similar profile to their normal-weight low-fit peers [121]. This suggests that high fitness levels may compensate the negative consequences attributed to body fat.

Unfortunately, in a large epidemiological study conducted in the USA, it was found that just 1 in 5 obese youth has healthy CRF [122]. Therefore, physical exercise programs aimed at improving CRF in this group of patients can be of enormous health interest. Among these programs, those that include high-intensity interval training have demonstrated an increased impact on youth’s CRF [123,124].

Considering the high prognostic power of CRF, the American Heart Association proposes to measure it as a vital sign, as is done with the assessment of other risk factors such as BP, tobacco use, alcohol consumption, blood glucose or blood lipid levels [125]. Another factor to be aware of is that overweight/obese youths may have some limitations in performing moderate and vigorous PA. In this context, personalized interventions should be designed according to the subject’s objective and up-to-date scientific knowledge. Therefore, the measurement of CRF in obese children and adolescents is not only of prognostic importance, but also allows for the personalization of the treatment according to the physical condition of each individual.

### 2.6. The Role of Tracking in Increased CV Risk in Adulthood

There is a strong correlation between childhood and adult obesity, and a large number of obese children transfer their adiposity into adulthood. Obese children and adolescents are five times more likely to become obese adults. About 55% of obese children continue being obese in adolescence, around 80% of obese adolescents will continue being obese in adulthood, particularly those suffering from severe obesity. About 70% of them will continue being obese over the age of 30 [126,127,128]. Age of the child, severity of obesity, and presence of parental obesity affect the tracking of obesity into adulthood. Most adolescents with obesity will continue being obese in adulthood, as persistence of obesity transfer into adulthood is associated with older age. In children under the age of 10, the risk of being obese is doubled if they have obese parents [129].

On the other hand, childhood and adolescent BMI is not a good predictor of adult obesity incidence. Only 20% of adults with obesity were obese as children or adolescents, and over 80% of obese people over the age of 30 were not obese as adolescents. Therefore, BMI has poor sensitivity to predict adult obesity [127]. Childhood obesity is not the only and primary factor that contributes to adult obesity. Adult obesity carries an increased risk of CVD. The link between obesity and CVD is explained by the CVD risk-factor profile that is often observed in obese adults. The profile includes increased rates of dyslipidemia, HTN, as well as T2D. Childhood obesity is a CVD risk-factor, and may lead to early atherosclerosis and premature CVD in adulthood. Even though CVD rarely manifests itself until adulthood, CVD risk factors have been observed in childhood [130]. HTN, dyslipidemia, impaired glucose metabolism, as well as systemic inflammation, have all been associated with vascular changes in childhood. If not adequately treated, they may contribute to an increased risk of adverse CV events in adulthood [131]. Nevertheless, it is important to determine what kind of independent effects childhood obesity has on CVD in adulthood. Many studies and meta-analyses have been conducted, all pointing to obese children being at higher risk for obesity as adults [132,133]. However, a large number of studies did not considered the effect of adulthood-incurred obesity, so it is impossible to form a precise conclusion of the relationship between childhood obesity and CV events in adulthood [134]. Studies that took into consideration the effect of obesity incurred in adulthood on CV events pointed to the fact that the effect of childhood obesity, as an independent factor, might not be great [135]. Those studies showing an association of obesity in childhood with CVD in adulthood, identified weight as a significant independent determinant [65].

However, there are other factors in favor of an association of early obesity with increased CVD risk:

Atherogenesis, a process leading to the development of atherosclerosis begins at an early stage of life [136]. Obesity in childhood accelerates this process and causes changes in blood vessels, especially in adolescence. The earliest sign of atherosclerosis is the appearance of fatty streaks, and atherosclerotic wall lesions are in direct connection to childhood obesity [136,137,138].

Clustering of CVD risk factors has been highly associated with obesity in childhood, including increased systolic blood pressure, elevated LDL-C, elevated TGs and reduced HDL-C [138,139].

Obesity with multiple CVD risk factors during adolescence is associated with an almost 15-fold increased risk of developing CVD before the age of 50 [140].

In an extensive study conducted on 276,000 children, Baker et al. note that an increased BMI in childhood correlates with the appearance of CVD in adults, and at the same time isn’t related to an increased BMI in adults [141].

BMI in late adolescence is directly related to atherosclerosis in middle age measured by coronary angiography—this relationship persisted even when BMI was adjusted for adults, as well as CVD risk factors [142].

Childhood obesity is a moderate risk-factor for adult obesity-related morbidity. However, the risk increase is not significant enough for childhood BMI to serve as a reliable predictor of the incidence of adult morbidities [132].

It is important to note, that the risks of T2DM, HTN, dyslipidemia, and carotid-artery atherosclerosis among overweight or obese children who became nonobese by adulthood were similar to those among persons who were never obese [143].

Table S1 summarizes the given evidence for tracking of CV risk factors from childhood to adulthood.

### 2.7. The Economic Impact of Childhood Obesity

A search in Pubmed found 264 articles published between 2001 and 2021 on the economic evaluation of prevention and treatment of childhood obesity and overweight. Out of them, 57 are cost-effectiveness studies of interventions aimed at reducing high BP among children and adolescents, or protocols for planned interventions with no results [144].

Two of the studies evaluated interventions to reduce obesity/overweight [145] and promote PA [146]. BP reduction was in both studies a secondary outcome. The Children’s Health Interventional Trial was an 11-month outpatient multidisciplinary family-based program implemented with 248 children with obesity or overweight in Germany [145]. The main focus was reducing weight, but the secondary objectives was the improvement of obesity-related health parameters as BP. The intervention obtained a reduction of systolic blood pressure (SBP) by −1.76 mmHg and diastolic blood pressure (DBP) by −2.82 mmHg. The program was cost-effective: on an aggregated level, future savings amounted to between €1859 and €1926 per person, and the return on investment was between 3.3% and 7.0%.

A school-based intervention study evaluated the effect of reduced salt intake among children and their families in China [147]. The focus was on BP of adults in the household rather than children. The intervention was very effective in lowering SBP in adults (−2.3 mmHg), but also in children, and even more in adults older than 60 years (−9.5 mmHg). It was also cost-effective (around $1358 per QALY [Quality adjusted life years] gained). Another study evaluated the cost-effectiveness of an early nutrition program—supplementing infant formula with long-chain polyunsaturated fatty acids—on health consequences in adulthood, more specifically high BP and the risk of HTN-related diseases in later life. The study results showed that the program is dominant (cost saving); it increases life expectancy by 1.2 QALY, with an incremental cost-effectiveness ratio (discounted to present value) of—€630.

Two of the most relevant studies perform an economic evaluation of screening and BP measuring of children and adolescents. The first one [148] compares costs and effectiveness of BP screening programs for adolescents in the US with population-wide preventive interventions, as reductions in salt intake or increasing physical education. Finding and treating the adolescents at highest risk (e.g., left ventricular hypertrophy) is the most cost-effective screening strategy with cost per QALY of $18,000 for boys and $47,000 for girls. Universal screening of all adolescents is dominated by specific population-wide strategies such as salt reduction (cost-saving [boys] and $650/QALY [girls]) and increasing physical education ($11,000/QALY [boys] and $35,000/QALY [girls]).

The second study [149] is a retrospective one that evaluated the initial use of ambulatory BP pressure monitoring for children with clinic BP measurements suggesting stage 1 HTN. It concludes that it is highly cost-effective (cost-savings in the long term of $2.4 million per 1000 patients).

In summary, the evidence so far has shown that some targeted interventions to prevent obesity and high BP in children and adolescents are potentially highly cost-effective.

### 2.8. Preventive Strategies for Hypertension in Children

There is limited literature regarding preventive strategies or intervention in children and adolescents with elevated blood pressure focusing on BP as the major end point [101,150]. In most cases, the main risk factor for HTN—increased body mass/fat mass is being targeted [2,101]. Similarly, there is limited evidence on population based primary prevention strategies in healthy children to reduce the future risk of HTN [2].

There are well established risk factors for developing HTN in children and adolescents. The major risk is overweight and obesity, additionally the nutritional scheme (quality and quantity of macronutrients), the amount of PA time (as a marker of CRF), parental-factors, sedentary/screen time and sleep time can independently increase prevalence of abnormal BP [151,152].

According to the Nuffield public health intervention ladder and it’s modifications, the interventions can be made on different level of individual or population impact [153]. Prevention strategies can be divided into three main groups: individual/family-based, local-community-based and nationally-based activities. Most of those actions are universal for all non-communicable diseases (NCD’s) or NCD’s risk factors.

Individual, family and school-based level interventions should be mainly focus on education on pro-health behaviors and building ability and capacity/consciousness to put PA and health-supporting diet as one of priorities. There is limited evidence on this in HTN yet similar activities are effective in increased body mass/fat interventions [154,155]. For example, as presented by Farpour-Lambert et al., even 3 months of regular PA can decrease SBP by 7–12 mmHg and DBP by 2–7 mmHg. Others reported similar or smaller effects of SBP/DBP reduction of 2–8 mmHg during different time of intervention or observation time [155,156,157,158].

On local/community level policy makers need to focus on the availability of healthy nutritional options, and the availability and places to perform PA (playing fields, recreational areas, biking lanes, etc.) [159]. Additionally, professional trainers support in different sports availability to children during/after school increases their PA hours during the week [160,161,162], as well as building availability for healthy food choices at schools by limiting vending machines, and improving quality and availability of healthy food at cafeterias/canteens [163,164,165,166].

National preventing strategies should focus on the availability of healthy nutritional choices, e.g., through taxation policies, products formulas modification (ex. reducing sodium) [167,168,169,170], education campaigns build and delivered for separate age groups, and supporting local authorities in building healthy environments [171,172].

The effectiveness of single strategies/activities is usually limited or low from the clinical perspective, yet addition of several multi-level activities can importantly influence the burden of CVD in children/youth as well as future costs—both health and economic [101,152,173].

## 3. Conclusions

Cardiovascular disorders with their origin in childhood obesity have multiple medical, social, and economic consequences that might be incurable at a later stage. Thus, the only effective strategy seems to be prevention.

Preventive strategies should include entire families and start ideally before conception. Families with obese members likely share not only genetic risks but also environmental and lifestyle-related exposures. The increased risk of obesity starts as early as before and during pregnancy. The results of numerous clinical trials indicate the influence of parental health on the development of the fetus and the risk of obesity in childhood and adolescence, while obesity and related metabolic risk factors are tracked to adulthood and increase the cardiovascular risk in the general population. Recent studies proved the role of childhood obesity-connected dyslipidemia, hyperinsulinism, or hypertension on increased CV risk. Some new risk factors like maternal smoking, postnatal growth patterns, the impact of diet components on gene expression or fructose intake on uric acid level, are also important elements. As the role of diet is complex, restriction of consumption of highly processed foods should be promoted and consumption of natural or minimally processed foods should be encouraged. Personalized interventions in improving cardiorespiratory fitness are recommended.

## Supplementary Materials

The following are available online at https://www.mdpi.com/article/10.3390/nu13114176/s1, Table S1: Evidence for tracking of CV risk factors from infancy and childhood to adulthood. Exemplary studies, reviews or meta-analyses are given for strength of evidence.
