# Supplementary material for: Obesity and Cardiometabolic Risk Factors: From Childhood to Adulthood
Source: Nutrients. 2021 Nov 22;13(11):4176. doi: 10.3390/nu13114176 (PMC8624977; doi:10.3390/nu13114176)
Supplement: Supplementary file 1 [file nutrients-13-04176-s001.zip › nutrients-1453133-supplementary.pdf]

Table S1: Evidence for tracking of CV risk factors from infancy and childhood to adulthood. Exemplary studies, reviews or meta-analyses are given for strength of evidence.

**CV risk factor**	**Evidence from**<br>type of study<br>(risk factor(s) studied)<br>*[reference]*	**Number of Subjects**<br>(or studies)	**Duration of follow-up**	**Tracking coefficient**<br>(95%CI)	**Interpretation of Tracking Coefficients / Study Conclusions**
**Maternal obesity**	Systematic review<br>(Maternal pre-pregnancy BMI or weight (MPBW))<br><br>*[Matern Child Nutr. 2018;14:e12561. https://doi.org/10.1111/mcn.12561]*	63,959<br>(16 studies)	up to 32 yrs	SBP:<br>-0.12 (-0.37, 0.14)<br>to 0.77 (0.27, 1.27)<br><br>DBP:<br>-0.003 (-0.23, 0.23) to 0.29 (0.05, 0.5)	*„… suggestive, but still limited, evidence for an association of MPBW with offspring's later blood pressure. However, offspring's anthropometric characteristics entirely explained the observed associations."*<br>*"MPBW could be an important, albeit indirect, determinant with respect to offspring's metabolic pathology. "*
	Meta-analysis<br>(Maternal pre-pregnancy BMI or gestational weight gain)<br><br>*[Matern Child Nutr. 2020;16:e13031. https://doi.org/10.1111/mcn.13031]*	12,406<br>(mother–offspring pairs)<br><br>(15 Studies)	up to 32 yrs	PPBMI and<br>insulin resistance:<br>0.063 (0.006, 0.121)<br><br>Weight gain and<br>insulin resistance:<br>0.028 (-0.012, 0.067)	*„... early-onset positive linear association between ppBMI and parameters indicating insulin resistance in the offspring even without manifest hyperglycaemia, but this effect might be indirect via offspring's actual anthropometry (body weight and adiposity)."*<br>*"suggestive, but still limited statistical evidence for a positive association of excessive GWG with offspring's insulin resistance. "*

**CV risk factor**	**Evidence from** type of study (risk factor(s) studied) *[reference]*	**Number of Subjects** (or studies)	**Duration of follow-up**	**Tracking coefficient** (95%CI)	**Interpretation of Tracking Coefficients / Study Conclusions**
**Gestational diabetes**	Systematic review and meta-analysis<br><br>*[Journal of Developmental Origins of Health and Disease 11: 599–616. doi: 10.1017/S2040174419000850]*	>30,000 (25 Studies)	up to 27 yrs	Data not given	*„... offspring exposed to gestational diabetes mellitus in utero demonstrate risk factors for CVD in childhood and adolescence, including elevated SBP, BMI z-score, and fasting plasma glucose that are evident from early life."*
**Maternal smoking**	Large prospective cohort study<br><br>*[International Journal of Obesity (2020) 44:1330–1340 https://doi.org/10.1038/s41366-019-0509-7]*	246,759	average 6.7 yrs	Data not given	*"Exposure to MS is associated with an increased risk of transition from low BW-to- adulthood obesity, and reduced likelihood of change from high BW-to-normal adult body weight."*
**Fetal growth** (Birth length, birth weight, duration of gestation)	Population based study<br><br>*[Am J Epidemiol 2001;154:21–9.]*	3,917	18 yrs	Data not given	*"Height in adolescence was predicted by length and weight at birth and by parents' height, whereas BMI was predicted by birth weight and parents' BMI. An especially high risk of overweight was found for subjects who were of normal length at birth but had a high birth weight and for those who had an overweight mother and/or father."*
**Artificial reproductive technologies** (e.g., IVF)	Ongoing studies				

**CV risk factor**	**Evidence from** type of study (risk factor(s) studied) *[reference]*	**Number of Subjects** (or studies)	**Duration of follow-up**	**Tracking coefficient** (95%CI)	**Interpretation of Tracking Coefficients / Study Conclusions**
**Serum urate**	Birth cohort study<br><br>*[American Journal of Hypertension 30(7) July 2017;* ***713*** *doi:10.1093/ajh/hpx037]*	449	3 yrs	Serum urate level (3 yrs) and serum urate levels<br><br>at 5 yrs of age:<br>0.33 (95%CI not given)<br><br>at 7 yrs of age:<br>0.20 (95%CI not given)	*"...significant evidence for tracking of uric acid from childhood into preadolescence."*
**Lipids**	Clinical trial<br><br>*[Lee et al. Lipids in Health and Disease (2017) 16:221 DOI 10.1186/s12944-017-0615-2]*	432	up to 21 yrs	TC: 0.58 (0.54, 0.63);<br>TGs:<br>0.39 (0.31, 0.48);<br>HDL-C: 0.51 (0.47, 0.56);<br>Non-HDL-C :<br>0.56 (0.52, 0.60);<br>LDL-C: 0.56 (0.52, 0.61);	*"The tracking patterns of blood lipids from adolescence to adulthood are notable."*
	Epidemiological study<br><br>*[Bogalusa Heart Study Am J Epidemiol 1991;133:884-99].*	1586	up to 12 yrs	TC: 0.38 to 0.66<br>TGs: 0.11 to 0.42<br>VLDL-C: 0.24 to 0.45<br>LDL-C: 0.44 to 0.69<br>HDL-C: 0.04 to 0.43<br>(95%CI not given)	*"Significant tracking of serum lipids and lipoproteins on a population basis."*
	Population-based study<br><br>*[The cardiovascular Risk in Young Finns Study Am J Epidemiol 1994;140:1096-110.]*	883	up to 12 yrs	TC: 0.48 to 0.58,<br>LDL-C: 0.53 to 0.58,<br>HDL-C: 0.53 to 0.58,<br>LDL/HDL-C ratio: 0.57 to 0.59,<br>TGs: 0.33 to 0.37<br>(95%CI not given)	*"Significant tracking was found in each of the serum lipid variables studied."*
**Cardiorespiratory fitness**	Clinical trial<br><br>https://doi.org/10.1002/ajhb.23381	142	up to 14 yrs	CRF 0.37 to 0.67	*"... all health-related physical fitness variables presented values considered moderate to high tracking"*

**CV risk factor**	**Evidence from** type of study (risk factor(s) studied) *[reference]*	**Number of Subjects** (or studies)	**Duration of follow-up**	**Tracking coefficient** (95%CI)	**Interpretation of Tracking Coefficients / Study Conclusions**
**Obesity/overweight**	Literature review<br>*[Obesity reviews 2008; 9:474-488]*	(25 studies)	up to 31 yrs	Data not given	*"The risk of overweight children to become overweight adults is at least twice as high compared with normotensive children.<br>Tracking of childhood weight status is moderate for overweight and obese youth."*
	Systematic review and meta-analysis<br>*[Obesity reviews 2016;79:95-107]*	>200,000 (15 Studies)	up to 35 yrs	Childhood obesity and obesity in adults:<br>5.21 (4.50, 6.02)	*"Obese children, and particularly obese adolescents, are likely to become obese adults."*<br><br>*Association between childhood and adolescent obesity and obesity in adults is strong.<br>However, childhood and adolescent BMI has poor sensitivity to predict adult obesity.*
	Meta-analysis<br>*[Umer et al. BMC Public Health (2017) 17:683 DOI 10.1186/s12889-017-4691-z]*	>40,000 (21 studies)	up to 50 yrs	Obesity (childhood to adulthood):<br>0.3 to 0.8<br>(mean 0.6, SD 0.1)<br><br>SBP: 0.11 (0.07, 0.14)<br>DBP: 0.11 (0.07, 0.14)<br>TGs: 0.08 (0.03, 0.13)	*"There is medium to strong tracking of adiposity across the life-span."*<br><br>*Childhood obesity may be a risk factor for selected adult CVD risk factors. Some outcomes were analyzed adjusted for adult adiposity.*

CV risk factor	**Evidence from** type of study (risk factor(s) studied) *[reference]*	**Number of Subjects** (or studies)	**Duration of follow-up**	**Tracking coefficient** (95%CI)	**Interpretation of Tracking Coefficients / Study Conclusions**
**Hypertension**	Systematic review / meta-analysis<br>*[Circulation. 2008 June 24; 117(25): 3171–3180. doi:10.1161/CIRCULATIONAHA.107.730 366].*	(50 studies) A total of 617 data points for SBP and 547 data points for DBP were used	up to 30 yrs	Meta-analysis<br>for 5 year follow up:<br>SBP: 0.43 (0.30, 0.59)<br>DBP: 0.32 (-0.16, 0.70)<br><br>for 10 year follow up:<br>SBP: 0.43 (0.27, 0.59)<br>DBP: 0.29 (0.17, 0.44)	*"Diverse populations show the evidence of BP tracking from childhood into adulthood is strong."*<br>*"There was a clear trend of weaker SBP and DBP tracking with longer follow- up."*
	Population-based study<br>*[International Journal of Hypertension, Volume 2018, Article ID 8429891, https://doi.org/10.1155/2018/8429891]*	KIGGS Study 2542 subjects	6 yrs	SBP 0.33 to 0.50<br>DBP 0.19 to 0.39<br>*(by sex and age groups)*<br><br>(95%CI not given)	*"...population-based data from Germany shows that BP in children and adolescents tracks only moderately over six years. BP in childhood is the strongest independent predictor of future BP."*

BMI – body mass index; BW – birth weight; CI – confidence interval; CRF – cardiorespiratory fitness; CV – cardiovascular; DBP – diastolic blood pressure; HDL-C – High density lipoprotein cholesterol; IVF – in vitro fertilization; LDL-C – low density lipoprotein cholesterol; MPPBMI – maternal pre-pregnancy BMI; MPPW – maternal pre-pregnancy weight; MS – maternal smoking; SBP – systolic blood pressure; SD – standard deviation; TC – total cholesterol; TGs – triglycerides; VLDL-C – very low density lipoprotein cholesterol;
